# Aortic pulse wave velocity in normals and heart failure patients

**DOI:** 10.1186/1532-429X-14-S1-P127

**Published:** 2012-02-01

**Authors:** Yi Wang, Jie J Cao, Yang Cheng, Nathaniel Reichek

**Affiliations:** 1 St. Francis Hospital, Roslyn, NY, USA; 2 SUNY, Stony Brook, NY, USA

## Summary

Aortic pulse wave velocity is altered by normal aging, as well as vessel wall pathology. We evaluated aortic compliance and its relationship to age in normals and patients with heart failure.

## Background

Aortic compliance (AC) can be evaluated noninvasively and its reduction with aortic pathology and age in normals has been demonstrated with both MRI and Doppler echo methods. Aortic pulse wave velocity (PWV), a measurement of the flow pulse traveling along aorta as a surrogate for AC, can be assessed using a single breath-hold phase contrast (PC) imaging technique. Congestive heart failure is often associated with a chronic cardiac remodeling process in which the myocardium either cannot eject blood very well (systolic heart failure); or the myocardium is stiff and ventricular chambers do not fill with blood easily (diastolic heart failure). We hypothesize that aortic stiffness is increased in the CHF population and its age dependency differs from that in normals.

## Methods

As normal controls, 196 healthy volunteers gave informed consent (96 male, age: 61.3±14.2) and were screened to exclude hypertension, hyperlipidemia and cardiovascular disease. Twenty two CHF patients had been referred for clinical CMR study with LVEF < 55%. Using the ‘candy cane’ view of aorta, an axial plane through the ascending and descending aorta at the pulmonary artery level was prescribed and a through-plane velocity encoded PC cine imaging was acquired with VENC of 150 cm/s, TR/TE/FA = 98ms/2.9ms/15° and voxel spatial resolution 1.3×2×6 mm3 on a 1.5T MRI scanner. The distance traveled by the aortic pulse wave, ΔD, was determined as the distance along the center line between the axial sections as imaged in the ‘candy cane’ image. For flow pulse onset, the cross correlation between the first halves of the ascending and descending aortic flow curves was calculated by varying the relative time shift between them. The Δt was the time shift at the maximal correlation. We then calculated PWV=ΔD/Δt. Linear regression was used to determine the relationships between PWV and age at both groups.

## Results

PWV in CHF and in normals correlate with age, as shown in the Figure as left graph and right graph of scatter plots, respectively. The linear regression in normals is: PWV (m/s) = -5.548 + 0.2601*Age; while in CHF patients: PWV (m/s) = -1.277 + 0.1879*age; However, the relationship is stronger in normals. Young and elderly patients with CHF had lower PWV than normals, likely due to reduced stroke volume and blood pressure in the CHF group.

**Figure 1:**
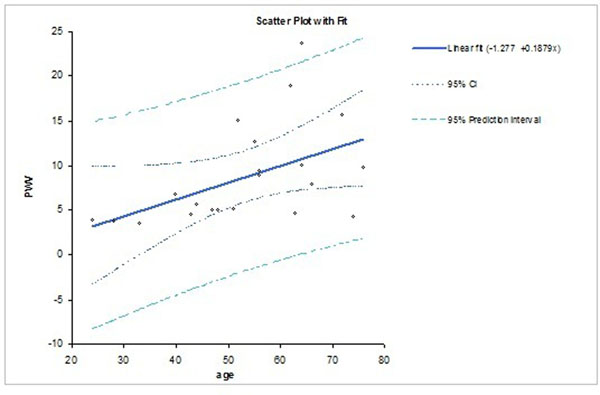
The scatter plot of the fitting curve between pulse wave velocity and age: CHF patients with R2=0.24, p=0.02, n=22.

**Figure 2:**
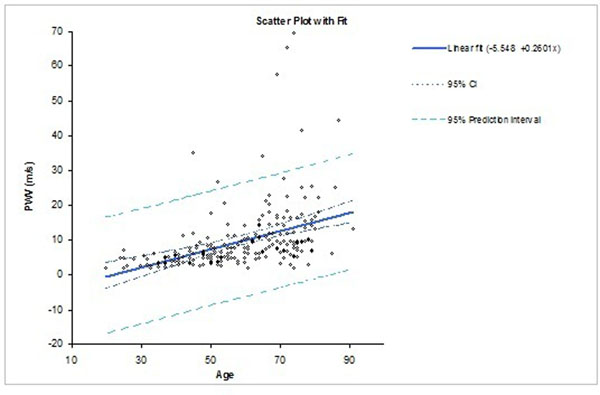
The scatter plot of the fitting curve between pulse wave velocity and age: normals with R2=0.18, p<0.01, n=196.

## Conclusions

Aortic stiffness increases with age in CHF patients, but the slope seems to differ from CHF patients to normals.

## Funding

None.

